# Characterising subtypes of hippocampal sclerosis and reorganization: correlation with pre and postoperative memory deficit

**DOI:** 10.1111/bpa.12514

**Published:** 2017-04-24

**Authors:** Anaclara Prada Jardim, Joan Liu, Jack Baber, Zuzanna Michalak, Cheryl Reeves, Matthew Ellis, Jan Novy, Jane de Tisi, Andrew McEvoy, Anna Miserocchi, Elza Marcia Targas Yacubian, Sanjay Sisodiya, Pamela Thompson, Maria Thom

**Affiliations:** ^1^ Department of Clinical and Experimental Epilepsy UCL Institute of Neurology Queen Square London WCN1BG UK; ^2^ Department of Neurology and Neurosurgery Universidade Federal de Sao Paulo, UNIFESP Sao Paulo Brazil; ^3^ Departments of Neuropathology National Hospital for Neurology and Neurosurgery Queen Square London WCN1BG UK; ^4^ Departments of Neurology National Hospital for Neurology and Neurosurgery Queen Square London WCN1BG UK; ^5^ Service de Neurologie, Département des Neurosciences Cliniques CHUV, University of Lausanne Switzerland; ^6^ Departments of Neurosurgery National Hospital for Neurology and Neurosurgery Queen Square London WCN1BG UK; ^7^ Departments of Neuropsychology National Hospital for Neurology and Neurosurgery Queen Square London WCN1BG UK; ^8^ Epilepsy Society, Epilepsy Society Research Centre Buckinghamshire SL9 0RJ UK

**Keywords:** hippocampal sclerosis, memory, mossy fiber sprouting, temporal lobe epilepsy

## Abstract

Neuropathological subtypes of hippocampal sclerosis (HS) in temporal lobe epilepsy (The 2013 International League Against Epilepsy classification) are based on the qualitative assessment of patterns of neuronal loss with NeuN. In practice, some cases appear indeterminate between type 1 (CA1 and CA4 loss) and type 2 HS (CA1 loss) and we predicted that MAP2 would enable a more stringent classification. HS subtypes, as well as the accompanying alteration of axonal networks, regenerative capacity and neurodegeneration have been previously correlated with outcome and memory deficits and may provide prognostic clinical information. We selected 92 cases: 52 type 1 HS, 15 type 2 HS, 18 indeterminate‐HS and 7 no‐HS. Quantitative analysis was carried out on NeuN and MAP2 stained sections and a labeling index (LI) calculated for six hippocampal subfields. We also evaluated hippocampal regenerative activity (MCM2, nestin, olig2, calbindin), degeneration (AT8/phosphorylated tau) and mossy‐fiber pathway re‐organization (ZnT3). Pathology measures were correlated with clinical epilepsy history, memory and naming test scores and postoperative outcomes, at 1 year following surgery. MAP2 LI in indeterminate‐HS was statistically similar to type 2 HS but this clustering was not shown with NeuN. Moderate verbal and visual memory deficits were noted in all HS types, including 54% and 69% of type 2 HS. Memory deficits correlated with several pathology factors including lower NeuN or MAP2 LI in CA4, CA1, dentate gyrus (DG) and subiculum and poor preservation of the mossy fiber pathway. Decline in memory at 1 year associated with AT8 labeling in the subiculum and DG but not HS type. We conclude that MAP2 is a helpful addition in the classification of HS in some cases. Classification of HS subtype, however, did not significantly correlate with outcome or pre‐ or postoperative memory dysfunction, which was associated with multiple pathology factors including hippocampal axonal pathways, regenerative capacity and degenerative changes.

## Introduction

The 2013 International League Against Epilepsy (ILAE) classification of hippocampal sclerosis (HS) in temporal lobe epilepsy (TLE) 8 was introduced to integrate previous terminology and provide a robust semiquantitative scoring scheme that could be of potential clinical and prognostic relevance to patients undergoing epilepsy surgery. It has subsequently been implemented in recent reported series of TLE patients 10, 12, 28, 33, 48. This classification includes the segregation of the common or typical pattern of HS (type 1, with neuronal loss in CA4 and CA1) from type 2 HS (neuronal loss restricted to CA1 subfield) and type 3 HS (neuronal loss restricted to CA4 subfield) based on NeuN stained sections. In practice, however, it can be difficult in some cases to distinguish type 1 from type 2 HS based on determining CA4 neuronal loss with nuclear NeuN immunolabeling.

Nevertheless, there is emerging data suggesting the ILAE classification system enables the identification of HS/TLE phenotypes that could account for clinical variability. HS subtypes may be predictive of seizure‐free outcomes following surgery 12, 28 and subtypes of HS and patterns of subfield neuronal loss have been associated with specific memory impairments, either pre‐ or postoperatively, that can occur with TLE. In particular, type 2 HS has been associated with preserved declarative memory prior to surgery 12, 33 although not in all surgical cohorts 48. These different findings between series may relate to the relatively smaller numbers of type 2 HS cases available for study. In addition, loss of dentate gyrus (DG) granule cells in TLE 12, 30 has also been linked with reduced preoperative memory capacity. There is marked plasticity and reorganization of the DG in HS/TLE exemplified by the reorganization of the mossy fiber pathway 40, alterations of hippocampal regenerative capacity 11, reduction of calbindin protein in granule cells 23, astroglial abnormalities 19, altered zinc signaling 38 as well as neurodegeneration including tau accumulation 22, all of potential relevance to the memory dysfunction in HS/TLE.

Our aim was to better characterize patterns of HS, associated regenerative and degenerative alterations and correlate these with preoperative memory function and postoperative outcomes including memory decline, in a large series of patients operated for TLE. We included cases that were either typical ILAE type 1 or type 2 HS as well as a group that we considered to have indeterminate patterns of sclerosis (between type 1 and 2), as judged by NeuN qualitative evaluation. MAP2 shows more confluent subfield immunolabeling in the normal CA4 of cell soma and dendritic networks. As the ILAE 2013 system is based on a qualitative assessment of overall NeuN labeling rather than actual neuronal cell counts, we hypothesized that MAP2 could provide a more sensitive qualitative evaluation in these indeterminate HS cases. A further aim was therefore to quantitate, compare and validate the usefulness of these markers for HS subtyping using a rapid automated analysis method.

## Methods

The cases were selected from the UCL Epilepsy Society Brain and Tissue Bank acquired over two decades (1994–2015). The study has ethical approval and patients consented for research. Applying current ILAE criteria for HS based on semiquantitative evaluation of NeuN staining 8, we included 52 cases with type 1, 15 cases with unequivocal type 2 HS and 18 indeterminate cases (Ind‐HS) where it was not possible to classify between type 1 and 2 HS based on qualitative evaluation of neuronal loss on NeuN in CA4 using ILAE criteria 8. The type 2 and Ind‐HS cases represented all cases available for study in our archive, cases only being excluded if there was a lack of consent for research or sufficient tissue was not available. In addition, we included seven TLE cases with No‐HS as a comparison for neuronal density measurements only. The clinical features of the HS groups are presented in Table 1 and the No‐HS cases in *Supporting Information* Table S1.

**Table 1:** Clinical features of ILAE subtypes compared to patients with indeterminate HS. Abbreviations: GS = secondary generalized seizures. *N* = the total number of cases in the group that data was available for.

Clinical group		TYPE 1 HS	TYPE 2 HS	IND‐HS
Number of cases		52	15	18
Gender	Male/Female	22/30	5/10	9/9
Age of onset		11.6 (1–41)	11.6 (0–21)	16.25 (9‐22)
Mean (range)				
years				
Age at surgery		35.6 (18–55)	35.4 (21–53)	27.34 (24–31)
Mean (range)				
years				
Side operated	Left/right	29/23	8/7	6/12
Seizure types (% of cases)	SPS	59.6	64.3	55.6
	CPS	94.2	92.9	100
	GS	78.8	85.7	72.2
IPI (% of cases)	Seizure	53.8 *	50	44.4**
	Head Injury	9.6	14.3	0
	Other	11.5	0	5.6
	None	21.2	35.7	44.4
Preoperative memory dysfunction (% of cases) *N* = number of cases with deficit/total tested	Moderate Verbal function deficit (L/R)	53%^†^	54%	33%
		(67%/36%)	(62%/40%)	(60%/20%)
		*N* = 26/49	*N* = 7/13	*N* = 5/15
	Severe Verbal function deficit	24.4%	23.1%	6.3%
		*N* = 11/45	*N* = 3/13	*N* = 1/16
	Moderate Visual function deficit (L/R)	50%^†^	69.2%	40%
		(46%/55%)	(62.5%/80%)	(40%/40%)
		*N =* 24/48	*N =* 9/13	*N =* 6/15
	Severe Visual function deficit	9.5%	33.3%	25%
		*N =* 2/21	*N =* 2/6	*N =* 1/4
	Moderate deficit in naming (L/R)	51.2%	76.9%	46.7%
		(58%/42%)	(100%/40%)	(100%/20%)
		*N =* 22/43	*N =* 10/13	*N =* 7/18
	Severe deficit in naming	27.9%	23.1%	33.3%
		*N =* 12/43	*N =* 3/13	*N =* 5/15
Postoperative memory decline in function (% of cases) *N* = number of cases with deficit/total tested	Decline in verbal function	22%	22%	27%
		*N =* 8/37	*N =* 2/9	*N =* 4/15
	Decline in visual function	13%	11%	13%
		*N =* 5/38	*N =* 1/9	*N =* 2/15
	Decline in naming	26%	10%	7.1%
		*N =* 10/38	*N =* 1/10	*N =* 1/14
Outcome SF (% of cases) (Number of cases)	1 year	69%	50%	56%
		(52)	(14)	(18)
	2 year	60%	36%	56%
		(50)	(11)	(18)
	5 year	66%	44%	58%
		(38)	(9)	(12)
	10 year	69%	43%	20%
		(13)	(7)	(5)

Initial precipitating injuries: “seizure” group includes a childhood seizure or Febrile Seizure; other category includes any other childhood event including episode of encephalitis/meningitis.

In 3.8%* and 5.6%** of these cases indicated more than one type of IPI was reported. Partial seizures were grouped into simple (SPS) and complex partial (CPS) in this dataset. The postsurgical outcome was classified using the ILAE system and in this table grouped as seizure free (SF). There was no statistical difference between these clinical factors in the three selected groups.

^†^The type 1 HS cases were selected to include cases both with and without memory decline. For definitions of severe and moderate memory deficits, refer to the main text.

### Immunostaining

In each case a representative formalin‐fixed and paraffin‐embedded tissue block was selected from the hippocampal resection which showed maximal representation of all subfields including the DG. Sections were cut at 5 μm thickness and immunohistochemistry staining was carried out using an antibody panel (see *Supporting Information* Table S2 for antibodies, sources, dilutions and methods): MAP2, NeuN (neuronal loss), ZnT3 [for mossy fiber pathway sprouting 13] and AT8 (phosphorylated tau) was carried out on the entire HS series and calbindin, olig2, MCM2 and nestin markers (hippocampal reorganization/regeneration) were carried out on 40 type 1 HS cases which were selected to include equivalent numbers with or without a preoperative memory deficit to identify pathological alterations that could be predictive of memory loss.

### Quantitative analysis

All pathology measurements were carried out blinded to the cognitive data and details of the quantitative methods are summarized in Table 2. Semi‐quantitative scores were carried out by two observers with good agreement (Kappa index 0.8–0.9); for cases with disparity in the grade, the slide was reviewed by a third person and a consensus was achieved.

**Table 2:** Outline of the methods for quantitative and qualitative evaluation of each pathological feature in hippocampal sclerosis cases with specified immunomarkers.

Measurement	Method
NeuN and MAP2 hippocampal subfield analysis for neuronal loss	*Quantitative evaluation*: Sections scanned at x40 and digitized (Leica SCN400 scanner, Leica Microsystems, UK) Six regions of interest (ROIs) were manually defined using Definiens Developer XD 64 software (Definiens AG Munich, Germany): dentate gyrus, subiculum, CA4*, CA3, CA2 and CA1^†^ Definiens software was trained to automatically detect immunostained structures corresponding to (i) neuronal nuclei (NeuN) or (ii) neuronal cell body and dendrites (MAP2) Labeling index (LI) [percentage of immunostained area/field fraction^‡^] for each subfield calculated
MAP2 labeling of basal dendrites on granule cells	*Semiquantitatively scored*: Rare dendrites Moderate numbers of granule cells with basal dendrites Many/majority of granule cells have basal dendrites
Olig2 and MCM2 in dentate gyrus	*Quantitative evaluation*: Section tiled at ×2 magnification (Nikon eclipse microscope) using Image Pro Plus (Media Cybernetics, Cambridge, UK) The dentate gyrus was outlined and images captured at ×40 representing this entire area (mean 55 fields/case; range 23–111) Positively labeled nuclei were tagged and the mean number of cells/μm^2^ calculated
Nestin immunolabeling	*Quantitative evaluation*: Slides scanned as for NeuN (above) Using Definiens software, the LI was quantified in four nonoverlapping ROI: granule cell layer, subgranular zone, CA4 and CA1
ZnT3 evaluation for Mossy fiber pathway sprouting	*Semiquantitatively scored*: Mossy fiber pathway sprouting in the molecular layer was semiquantified using a three‐point scale: 0: no sprouting/labeling 1: weak or focal labeling 2: intense labeling The presence of a residual mossy fiber pathway^§^: 0–2 (as above) Axonal sprouting in the subgranular zone: 0–2 (as above)
Calbindin in granule cell layer	*Semiquantitatively scored*: 0: total loss/absent expression in granule cells 1: severe loss/rare labeled granule cells 2: partial loss/approximately half granule cells labeled^¶^ 3: retained/virtually all granule cells labeled
AT8 labeling for hippocampal phosphorylated tau	*Semiquantitatively scored*: Six point “modified Braak” tau scoring scale, in any hippocampal subfield, dentate gyrus, subiculum, alveus/white matter: 0: negative 1: rare grains 2: rare threads 3: few grains or threads 4: moderate neuropil threads/neuronal labeling 5: marked deposition**

*For CA4 ROI care was taken to exclude the basal dendrite zone of the granule cells in MAP2 sections.

^†^In the majority of cases all six regions were acquired.

^‡^Labeling index (LI) refers to the percentage/index of immunostained area, that is, the fraction of each digitized high power field that is immunolabeled with an intensity above the set threshold.

^§^Synaptic‐like positive labeling in CA4 and CA3.

^¶^The pattern of positive labeling restricted to the dispersed granule cells only, as previously reported in HS 2, 26 was also noted if present or not.

**AT8 labeling of level 5 was equivalent to Braak stage IV in hippocampus.

### Cognitive measures

Preoperative cognitive data was compared to retest data at 1 year following surgery, as previously described 43 and were available for the majority of patients from routine surgical evaluations undertaken at the National Hospital. Memory was assessed with the List Learning and Design Learning subtests from the Adult Memory & Information Processing Battery prior to 2007 and its successor the Brain Injury Rehabilitation Trust (BIRT) Memory and Information Processing Battery from 2007. These measures have previously been shown to be sensitive to hippocampal pathology 1. Naming capacity was assessed using the graded naming test (GNT) 5, a measure sensitive to dominant temporal lobe resections 9. Preoperatively patients were classified as having a moderate verbal or visual memory deficit if they scored 1 SD or more below average, and as having a severe deficit if they scored 2 SD or more below average. A patient was classified as having experienced a postoperative decline in verbal memory, visual memory and GNT capacity if their postoperative scores declined more than would be expected from retesting on the basis of the reliable change indices (with a confidence interval of 90%) 6.

### Clinical data and statistical methods

The duration and age at onset of epilepsy, any initial precipitating injuries (IPI) and outcomes following surgery were recorded. Statistical analysis was carried out between HS groups, pathology measures, psychometric and clinical data using SPSS (version 21 for windows) and included nonparametric tests (Mann–Whitney and Kruskall–Wallis tests) between HS groups and the MANOVA for multivariate analysis of factors in relation to psychometric categories; *P* values of <0.05 were regarded as significant.

## Results

### Application of MAP2 in classifying indeterminate HS subtypes

**Figure 1:**
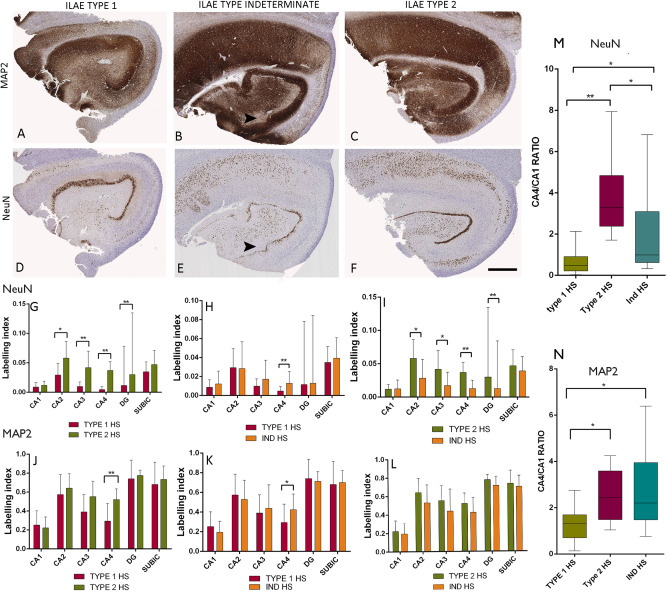
*Hippocampal sclerosis (HS) patterns with NeuN and MAP2*. Comparison of labeling in type 1 HS (**A,D**), type 2 HS (**C,F**) and indeterminate HS (Ind‐HS) (**B,E**) for MAP2 (**A,B,C**) and NeuN (**D,E,F**). MAP2 and NeuN both clearly demonstrated the neuronal loss in CA1 in all HS subtypes. Arrow heads indicate regions with patchy neuronal loss in CA4 and the hilus in Ind‐HS, particularly in the subgranular zone, but with overall strong CA4 labeling in MAP2 (**B**). **G–L.** Bar graphs of the labeling index (LI) for NeuN (**G,H,I**) and MAP2 (**J,K,L**) between subtypes of HS. **G**. There was a significant difference in NeuN LI between type 1 and type 2 HS cases in this series in all subfields except for CA1 and the subiculum confirming the distinct patterns of neuronal loss (*P* < 0.001 to *P* < 0.0001). **H.** Comparison of type 1 and Ind‐HS with NeuN showed significant differences in the LI in only CA4 (*P* < 0.0001) whereas **I** between type 2 and Ind‐HS, differences were observed for all subfields except CA1 and the subiculum (*P* < 0.006 to *P* < 0.0001). **J**. MAP2 LI also showed significant differences between type 1 and type 2 for CA4 (*P* < 0.0001). **K.** Comparison of type 1 and Ind‐HS, with MAP2 the LI showed differences in CA4 (*P* = 0.01) whereas (**L**) there were no significant differences between type 2 and Ind‐HS cases for any subfield on MAP2. **M.** Box plots of mean CA4/CA1 ratios for NeuN are highest in type 2 HS with significant differences between all three groups whereas MAP2 classifies Ind‐HS as similar to type 2 HS. Statistical differences are shown as (*P < 0.01–0.001, **P < 0.0001). The values for the dentate gyrus (DG), labeling index in G to I are shown as ×10^−1^ for presentation purposes. Bar for **A** to **F** is 1 mm.

MAP2 highlighted better preservation of CA4 neuronal and dendritic labeling in Ind‐HS compared to type 1 HS cases (Figure 1A,B) with comparable intensity to type 2 HS (Figure 1C). By comparison, with NeuN labeling the CA4 neuronal densities in Ind‐HS cases appeared intermediate between type 1 and 2 on qualitative assessment alone (Figure 1D–F). Quantitative and statistical analysis with whole slide scanning analysis confirmed that with NeuN, CA4 LI in Ind‐HS was between type 1 and 2 HS and statistically different from both (*P* < 0.0001) (Figure 1G–I). With MAP2, however, CA4 LI in Ind‐HS cases was not statistically different to type 2 HS (*P* = 0.07) but different from type 1 HS (*P* = 0.01) (Figure 1J–L). Furthermore, compared to LI in No‐HS cases, there was a mean relative reduction of MAP2 LI in CA4 of 36% in type 1 HS compared to only 8% in Ind‐HS and 0% in type 2 HS. Ratios of CA4/CA1 LI for NeuN (Figure 1M) and MAP2 (Figure 1N) also confirmed the utility of MAP2 in statistically classifying Ind‐HS as similar to type 2 HS.

### Cognitive performance in relation to HS type and hippocampal neuronal loss

#### Moderate deficit

Cases with moderate preoperative verbal, visual memory and GNT deficits were represented in both type 2 and Ind‐HS groups (in addition to the selected type 1 HS group) (Table 1); although deficits were noted more frequently in type 2 than Ind‐HS for all three domains, there were no significant differences between these two groups. Moderate deficits in verbal memory were more common in left than right sided resections (63% vs. 37%) (*P* = 0.004) and similarly for GNT deficits (65% vs. 35%) (*P* = 0.002) over all cases. A significant left sided predominance in cases with GNT deficits was also noted in type 2 HS (*P* = 0.016) and Ind‐HS (*P* = 0.013) with all left sided resections in these HS groups showing this deficit (Table 1).

#### Severe deficit

Cases with severe preoperative verbal, visual memory and GNT deficits were represented in both type 2 and Ind‐HS groups, but with no significant difference between groups (Table 1).

#### Cognitive decline

Verbal memory decline was present in 32% (14/61), visual memory decline in 13% (8/62) and a decline in naming in 19% (12/62) over all HS cases that had retesting 1 year postoperatively; cases with memory decline in each domain were represented in type 1, type 2 and Ind‐HS with no significant differences between groups (Table 1).

Comparison with pathology measures showed significant associations between lower mean NeuN or MAP2 LI in CA1, CA4, DG and subiculum subfields with moderate or severe preoperative memory deficits in all cases or HS subtypes as summarized in Table 3. Multivariate analysis showed a significant association of these pathology variables with moderate verbal memory deficits. For memory decline, the only significant observation was higher mean MAP2 LI in the subiculum in cases with naming decline postoperatively (Table 3).

**Table 3:** Results of statistical analysis between pathology measures and memory deficits. Abbreviation: MRA = multiple regression analysis performed with SPSS to predict the effect of the multiple variables on the memory deficit.

Cognitive domain	Subfield	Pathology measurement	Mean LI/value* in cases with deficit/decline (SD) *N =* number of cases	Mean LI/value* in cases without deficit/decline (SD) *N* = number of cases	Significance
Verbal memory deficit (moderate)	CA1	NeuN LI	0.008 (0.006) *N =* 35	0.01 (0.008) *N* = 40	*P* = 0.04 (all HS)
			0.007 (0.004) *N* = 24	0.012 (0.01) N = 22	*P* = 0.019 (type 1 HS)
	CA4	MAP2 LI	0.3 (0.2) *N =* 37	0.4 (0.18) *N =* 35	*P* = 0.04 (all HS)
			0.26 (0.16) *N =* 25	0.37 (0.2) *N =* 20	*P* = 0.05 (type 1 HS)
	DG	Basal dendrites*	1.51 (0.73) *N =* 37	1.92 (0.8) *N =* 38	*P* = 0.025 (all HS)
	MRA	All above			*P* = 0.016 (*R* ^2^ = 0.15) (all HS)
Visual memory deficit (moderate)	CA1	MAP2 LI	0.2 (0.12) *N =* 31	0.3 (0.15) *N =* 36	*P* = 0.042
Naming deficit (moderate)	DG	NeuN LI	0.1 (0.06) *N =* 20	0.14 (0.07) *N =* 20	*P* = 0.03 (Type 1 HS)
			0.25 (0.09) *N =* 10	0.38 (0.03) *N =* 3	*P* = 0.02 (Type 2 HS)
	CA4	MAP2 LI	0.31 (0.05) *N =* 7	0.5 (0.15) *N =* 6	*P* = 0.008 (Ind‐HS)
	MRA	All above			Not significant (all HS); *P* = 0.03 (*R* ^2^ = 0.47) (Ind‐HS)
Verbal memory deficit (severe)	DG	Residual MFP*	1.27 (0.6) *N =* 15	1.56 (0.6) *N =* 57	*P* = 0.013 (all HS^†^)
Visual memory deficit (severe)	Subiculum	MAP2 LI	0.7 (0.06) *N =* 5	0.83 (0.08) *N =* 22	*P* = 0.008 (all HS^†^)
Naming deficit (severe)	DG	NeuN LI	0.12 (0.08) *N =* 20	0.16 (0.09) *N =* 50	*P* = 0.05 (all HS^†^)
Verbal memory decline	None				
Visual memory decline	CA4	Nestin LI	0.23 (0.06) *N =* 3	0.1 (0.06) *N =* 24	*P* = 0.016
Naming decline	Subiculum	MAP2 LI	0.78 (0.13) *N =* 11	0.64 (0.21) *N =* 43	*P* = 0.03 (all HS)
		AT8*	55% *N =* 6/11	22% *N =* 10/45	*P* = 0.035 (all HS)
	DG	AT8*	45.5% *N =* 5/11	11% *N =* 5/45	*P* = 0.008 (all HS)
	MRA	All above			*P* = 0.008 (*R* ^2^ = 0.2)

Pathology factors showing significant differences between the presence or absence of deficits in each memory domain are listed and the mean values shown, including the labeling index (LI) of immunostaining for NeuN or MAP2 (except for pathology factors indicated with an asterisk, where the semiquantitative score scales are detailed in supplementary methods).

^†^In cases with the severe memory deficits it was not possible to analyze data further for HS subtypes be caused by the small numbers with a severe deficit in each group.

### DG pathology in HS types and cognitive measures

#### ZnT3

**Figure 2:**
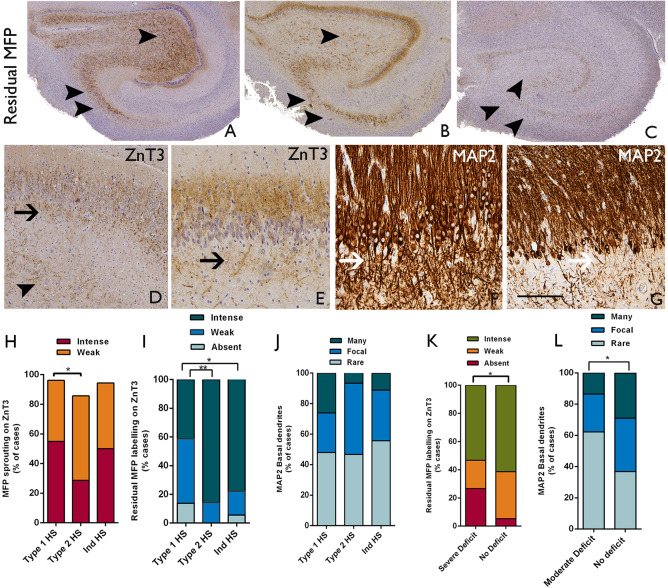
*Mossy fiber pathway, granule cell basal dendrites in relation to hippocampal sclerosis (HS) type and memory function*. **A–E**. ZnT3; **F–G.** MAP2. **A.** Intense labeling of a retained or normal mossy fiber pathway (MFP) trajectory is shown and absent sprouting. **B**. shows moderate labeling of the normal MPF as well as sprouting in the molecular layer and in **C** the pathway is indistinct (the two arrowheads indicate CA3 and one arrowhead CA4 in the MFP in each figure). **D**. ZnT3 labeling in the subgranular zone (SGZ) is present (arrow) with weak MFP sprouting in the molecular layer and a weak residual MFP in CA4 (arrowhead). **E**. shows more intense MFP sprouting in the molecular layer with ZnT3 also showing some sprouted fibers in the SGZ (arrow). **F.** Basal dendrites on granule cells are highlighted with MAP2 and in this case, are very numerous (arrows) forming a mesh of processes in the SGZ. **G.** In other cases, rarer granule cells (arrow) are observed to have basal dendrites. **H**. Bar chart of the presence of MFP sprouting in the molecular layer between HS types showing differences between type 1 and type 2 HS (*P = 0.05). **I**. The presence of a better preserved or residual MFP also showed significant differences between HS groups with better preservation in non‐type 1 HS cases (*P = 0.01, **P = 0.003). **J**. The presence and density of basal dendrites on granule cells showed some variation between HS groups, but the differences were not significant. (Of note in the three bar graphs **H** to **J**, the values for Ind‐HS group are always between observed values for type 1 and type 2 HS). **K**. In all HS/TLE cases, the presence of a better preserved or residual MFP (weak + intense) was associated with a lack of severe preoperative verbal memory deficit (*P = 0.013). **L**. The presence of basal dendrites in granule cells was associated with the lack of moderate verbal memory deficit (*P = 0.025). Bar is equivalent to approximately 1 mm in **A** to **C**, 100 microns in **D** and **E** and 50 μm in **F** and **G**.

Mossy fiber pathway sprouting with ZnT3 was observed in all HS types (Figure 2D,E). Intense ZnT3 mossy fiber pathway sprouting was more frequently observed in type 1 than type 2 HS cases (55% vs. 29%, respectively) with a trend for a significant difference between these groups (*P* = 0.05) (Figure 2H); there were no differences in mossy fiber pathway sprouting patterns between type 1 and Ind‐HS groups. A residual mossy fiber pathway was significantly better preserved in both type 2 HS (*P* = 0.003) and Ind‐HS cases (*P* = 0.01) than type 1 HS (Figure 2A–C,I). Labeling of sprouted fibers in the subgranular zone with ZnT3 (Figure 2D,E) was more prevalent in type 2 than type 1 HS (*P* = 0.01). The presence of basal dendrites on granule cells as visualized with MAP2 varied dramatically between HS cases (Figure 2F,G); although more prevalent in type 1 HS the presence of basal dendrites was not significantly different from type 2 and Ind‐HS based on semiquantitative scores (Figure 2I). There was a strong positive correlation in all HS cases between the presence of basal dendrites on granule cells and ZnT3 sprouted fibers in the subgranular zone (*P* < 0.0001). Statistical analysis showed an association between severe preoperative verbal memory deficit and lack of a preserved mossy fiber pathway (Table 3, Figure 2K) and moderate preoperative verbal memory deficit and the presence of basal dendrites on granule cells (Table 3, Figure 2L).

#### Calbindin

**Figure 3:**
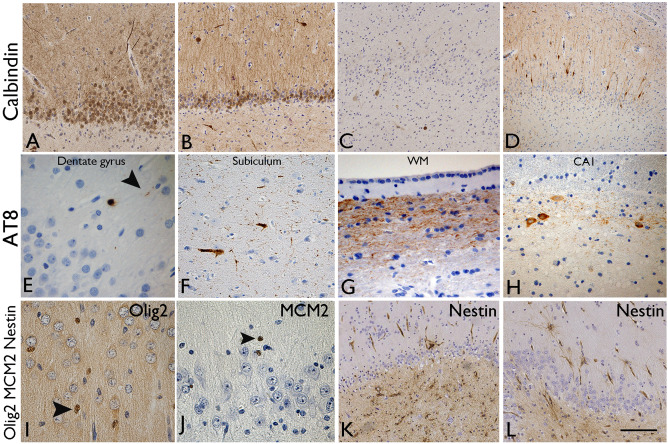
*Dentate granule cell layer: evidence of regenerative and degenerative pathology changes*. **A–D.** Calbindin immunohistochemistry varied between cases and was semiquantified as **A.** Preserved expression in granule cells and their apical dendrites, **B.** Partial loss of expression in approximately half of granule cells, **C.** Virtual total loss of expression with only interneurons in CA4 showing positivity. **D**. A frequent pattern in HS/TLE is calbindin expression in the dispersed or migrated cell types whereas the basal granule cells are calbindin negative. There was no statistical association between calbindin expression patterns and memory deficit. **E**–**H** AT8/phosphorylated tau immunohistochemistry, common patterns were **E.** Occasional grains and threads in the molecular layer, **F.** More frequent threads and positive neurones in the subiculum, **G.** Labeling of axons in the hippocampal white matter, **H.** Tau positive neurones in the margins of CA1 subfield: The presence of AT8 in the dentate gyrus and subiculum was significantly associated with memory decline postoperatively. **I.** Olig2 immunohistochemistry: Distinct labeling of a proportion of small nuclei in the dentate gyrus, some in a satellite position in relation to the granule cells. **J.** MCM2 immunohistochemistry: Less frequent, small immature nuclei were present through the dentate gyrus but no labeling of mature granule cells was seen. The density of olig2 and MCM2 positive cells declined, but not significantly, with memory deficit. **K**–**L**. Nestin immunohistochemistry: **K.** Labeling of multipolar cells was most prominent in the subgranular zone and in CA4; **L.** Illustration of occasional nestin‐expressing cells in the molecular layer. There was a positive correlation between nestin labeling in CA4 in patients with visual memory decline. Bar is equivalent to approximately 100 μm in **A** to **D**, 20 μm in **E**, **G**–**J** and 75 μm in **F**, **K** and **L**.

Total loss of calbindin expression in granule cells was noted in 35%, severe loss in 32.5%, partial loss in 30% and no loss in 2.5% of all cases (Figure 3A–C). The pattern of restricted calbindin expression in dispersed granule cells only was present in 30% (Figure 3D) 2, 26 which significantly correlated with MAP2 basal dendrites on granule cells (*P* = 0.002) and ZnT3 in the subgranular zone (*P* = 0.001). There were no statistical associations between calbindin expression patterns and memory deficits (*Supporting Information* Table S3).

#### AT8

In 35% of cases there was no evidence of hippocampal phosphorylated tau (score 0). In 53.3% of cases rare grains or threads were noted (score 1 and 2) (Figure 3E) with a few AT8 threads in 10.4% (score 3) and moderate AT8‐positive tau load in 1.3% of cases (score 4); there was no case with a score 5. AT8 was present in the subiculum (47%) (Figure 3F), the DG molecular layer (37%) (Figure 3E) and axonal‐like fibers in the parahippocampal gyrus white matter (20%) (Figure 3G); labeling was noted in the alveus in 9 cases and in 10 cases prominent labeling of horizontal neurones and fibers in the outer part of CA1 pyramidal cell layer (and CA2) was noted (Figure 3H). The presence of AT8 in the DG and subiculum was significantly associated with naming decline 1 year postoperatively (Table 3).

#### Olig2 and MCM2

Labeling was restricted to the nuclei of small, immature, round to ovoid cells in the DG (Figure 3I,J); some were occasionally noted in a “satellite” position in relation to mature granule cell neurones. The density of olig2 and MCM2 positive cells were lower in HS cases with severe preoperative verbal and visual memory function but not significantly different to cases without deficit (*Supporting Information* Table S3).

#### Nestin

Expression in the DG, apart from in the endothelium, was virtually restricted to multipolar cells in the subgranular zone and more prominently in CA4 (Figure 3K); occasional multipolar cells in the molecular layer were seen in some cases (Figure 3L). There was a positive correlation between nestin LI in CA4 in patients with visual memory decline at 1 year (*P* = 0.016) but not for other hippocampal ROI (Table 3).

### Clinical correlations and outcome

There was no significant difference in the epilepsy history between type 1, 2 and Ind‐HS groups, in regard to age of onset and IPI history, although a lack of a reported IPI was more frequent in the atypical/non‐type 1 HS cases (Table 1). Complete seizure‐freedom was less frequent for type 2 compared to type 1 HS, with Ind‐HS cases falling between these two groups at each period of follow‐up from 1 to 5 years; however, these differences between the groups were not significant (Table 1). There was a correlation between the modified tau score (*R* ^2^ = 0.075) and age at surgery, but not with other clinical factors including age of onset of epilepsy or IPI type including history of head injury.

## Discussion

HS is the most frequent pathology in TLE and classification of subtypes is currently recommended by the ILAE as it may inform on different clinical syndromes, outcomes and co‐morbidities, including memory impairment 8. In this study, we have shown that additional quantitative evaluation of MAP2 enables the classification of equivocal or indeterminate cases, which seem to fall between type 1 and 2 HS based on current ILAE criteria, as being statistically similar to type 2 HS. Nevertheless, memory deficits present before surgery in our cohort do not align with one HS type but correlate with several pathology factors including neuronal loss in several subfields and mossy fiber reorganization. Postoperative memory decline was associated with neurodegenerative and regenerative pathological alterations. These findings suggest that multifactorial patho‐mechanisms could be operational in the memory impairments associated with HS/TLE.

MAP2 highlights both dendritic labeling as well as the neuronal soma, and is confirmed in this study as a useful adjunct to NeuN for the more sensitive evaluation of neuronal preservation in equivocal cases, particularly in subfields as CA4 with lower neuronal densities. We propose that introduction of MAP2 staining in addition to NeuN in equivocal cases can help in routine evaluation of sclerosis patterns. Qualitative impressions of MAP2 were supported by quantitative analysis in this study employing whole slide scanning image analysis systems which are being increasingly applied in routine diagnostic practice 24. This technique has advantages over both subjective semiquantitative as well as other quantitative methods 12, 41, as the entire hippocampus is evaluated and rapidly, automatically analyzed in an unbiased fashion. Based on MAP2 quantitative analysis, Ind‐HS were statistically similar to type 2 HS whereas with NeuN quantitation they fell between type 1 and 2 HS. Interestingly, mossy fiber pathway reorganization which differed between 1 and 2 HS groups was intermediate in Ind‐HS cases which could suggest a pathological continuum in the process of HS. Furthermore, previous studies have reported fewer seizure‐free outcomes following temporal lobe resections in ILAE type 2 compared to type 1 HS 7, 14, 28. In our series, which included a large number of ILAE type 2 cases, lower rates of seizure‐free outcomes were also noted and although not statistically different from type 1 HS cases, the outcomes for the Ind‐HS group were again noted to be intermediate between type 1 and 2.

The hippocampus has a central role in episodic memory 35 and memory impairment frequently accompanies HS 18, 20 with a dominant role for the left intact hippocampus in verbal memory and the right with visual‐spatial memory, as also reflected in this current series. Temporal lobe surgery has offered a unique opportunity to correlate pathology, in particular the severity and distribution of hippocampal subfield neuronal loss and gliosis, with any memory dysfunction, in an aim to elucidate normal mnemonic pathways and networks and their potential disruption and reorganization in epilepsy 3, 10, 12, 30, 46. Previous quantitative studies have reported a correlation between reduced left CA1 neuronal densities and preoperative verbal memory deficits 3, a pre‐eminent role for the loss of granule cells and memory dysfunction 12, 30 while other studies showed a correlation with overall neuronal loss across hippocampal subfields 46. In the present study, we have shown correlations between neuronal loss assessed with whole slide scanning on NeuN and MAP2 in DG, CA1, CA4 and subiculum subfields and moderate to severe preoperative memory dysfunction. We failed to confirm loss restricted to a single subfield associating with either a memory deficit or decline and multivariate analysis also supported a synergistic contribution of multiple pathology factors to memory dysfunction.

Recent studies have also implicated associations between ILAE HS type and memory function. In a series of 13 patients with ILAE type 2 HS, a lack of dysfunction in declarative memory capacity, as assessed by intracarotid amobarbital (WADA) testing in addition to verbal memory tests, was observed; this suggested functional integrity of hippocampal memory networks despite neuronal depletion of the CA1 sector 10, 12. This finding was supported by a subsequent report of 36 HS/TLE patients in which type 2 HS cases were over‐represented in patients with normal memory scores 33 although disputed in a further report of six type 2 HS cases who all had impaired memory function 48. A “subordinate” role of CA1 in hippocampal memory circuitry is thus still open to debate. The relatively small number of type 2 cases in each of these series might explain these differences reported as well as differences in memory test designs. In our series, only two patients had had a WADA test, a procedure no longer used at our institute to assess memory function. We included all the available cases of type 2 HS from our institute and confirmed preoperative memory deficits were frequent for visual and verbal memory domains with the anticipated left vs. right lateralization patterns. Our findings support that intact memory function is not always present in type 2 HS.

ZnT3 has been recently employed as a robust marker of the mossy fiber pathway and its re‐organization in epilepsy 13. Zinc is also known to have important modulatory effects on synaptic transmission 25 and a role of ZnT3 in cognitive impairment in neurodegenerative diseases has been proposed 39, 45. Using this marker in our series, mossy fiber pathway sprouting was a common finding with some differences between HS groups including a more prominent residual mossy fiber pathway in type 2 and Ind‐HS than type 1 HS. Furthermore, there was an association between loss of the normal mossy fiber pathway and preoperative verbal memory deficit, suggesting that integrity of this anatomical pathway is involved in functional memory circuits. Coras *et al*, also noted better anatomical preservation of CA4/3 myelinated fibers connecting to the fimbria and subiculum in HS type 2 patients with intact memory functions 12. We also noted a great variability in the density of basal dendrites on granule cells between HS cases as highlighted with MAP2. It is known that the number of basal dendrites are increased in HS/TLE and were present in 40% of granule cells in a previous Golgi study 44. Similarly, in experimental models, basal dendrites are a feature of immature granule cells, their numbers increase following seizures and they are innervated by mossy fibers and potentially contribute to recurrent excitatory circuits 29. We noted that the presence of basal dendrites strongly correlated with ZnT3 labeling of mossy fibers in the subgranular zone, which is in keeping with these experimental findings. Our observation of lower densities of basal dendrites on granule cells in patients with preoperative memory deficit could also imply their contribution to anatomical memory circuits in the damaged hippocampus. In addition, basal dendrites as a surrogate marker of newly generated or immature granule cells could support the concept of an impaired regenerative capacity/plasticity in HS patients with memory deficits.

The subgranular zone is one of the main regenerative sites in the adult human brain with estimates of 700 new cells added per day 34. Seizures are known to influence progenitor cell turnover 29 and previous in‐vitro studies on human epilepsy tissues have correlated loss of granule cell proliferative capacity with memory dysfunction 11. In the current series, we selected equal numbers of classical or type 1 HS both with and without memory deficits in an aim to tease out any pathological differences in DG regenerative capacity that could be measured in fixed tissue sections. We utilized cell cycle marker MCM2 to measure overall cell replicative capacity in the hippocampus and olig2 as a marker of oligodendrocyte progenitor cells, previously shown to be the largest population of proliferating cells in tissues from focal epilepsy 16. Calbindin is not expressed in immature granule cells and reduced calbindin expression in granule cells is a frequent observation in TLE, particularly in less mature basal granule cells 1, 2, 26. Furthermore, lower calbindin levels in granule cells have been postulated to influence memory capacity, being depleted in Alzheimer's disease 36 and a loss of calbindin has been previously shown in TLE to correlate with verbal memory dysfunction 23. In this current study, although differences were noted with our methods, we were unable to confirm a significant correlation between olig2, MCM2 and calbindin labeling with pre‐ or postoperative memory dysfunction in type 1 HS.

Further decline in memory following temporal lobe surgery is reported to occur in up to 25%, with older patients being particularly vulnerable 21, 43. A better‐preserved hippocampus has been shown to be predictive of greater postoperative decline in verbal memory function 47 and more significant postoperative memory declines have been reported with ILAE type 2 HS 12 or better preserved CA1 3. In terms of quantitative confirmation of regional neuronal preservation in the present study, we identified a statistical association between MAP2 labeling only in the subiculum and naming decline at 1 year. However, we also identified an association between the presence of phosphorylated tau pathology in the subiculum and DG and a postoperative decline in naming. The tau load was overall low in this predominantly young adult cohort but correlated with age at surgery and is unlikely to represent either a primary tauopathy or be relevant to the cause of epilepsy. Previous surgical 31, 37 as well as post‐mortem studies in long‐term epilepsy 42 support an age‐accelerated deposition of tau, with some patterns potentially related to brain injury. Enhanced accumulation of p‐tau has been shown in the molecular layer, granule cells and mossy fibers in association with seizures in Alzheimer's disease transgenic mouse models 49 and tau accumulation is modulated by synaptic activity in experimental systems 37. We have also previously shown “epilepsy‐specific” patterns of tau accumulation in the hippocampus in some patients over 50 years old at surgery, including early involvement of the DG 37. This study therefore highlights the potential vulnerability of some younger adults with epilepsy to a secondary tauopathy, which may predispose to postoperative memory decline following surgery.

Reactive astrogliosis is a prominent component of HS and plasticity of glial cell populations and functional changes are considered to contribute to disease mechanisms in epilepsy 4, 44 including potential effects on memory 19, 32. Glial cells exceed brain cells in their complexity, diversity and number, with roles for glial transmitters in memory, including long term memory consolidation 15, 27 and spatial memory 17. Previous studies addressing glial populations in relation to memory in HS/TLE have utilized GFAP 3 and showed no correlation between glial density in any subfield and preoperative neuropsychology test scores. Nestin selectively labels a subset of immature glial cells in the subgranular zone of the hippocampus which contribute to adult neurogenesis 37; it is also an intermediate filament that is transiently re‐expressed in reactive, proliferating glia at injury sites. We observed that nestin‐positive glia in type 1 HS had restricted distribution and were more prominent in hilar subgranular zone in HS cases; furthermore, their density was inversely linked to visual memory decline postoperatively.

The limitations of this study include the relatively small numbers of type 2 and Ind‐HS cases compared to type 1 HS cases. Although the surgical specimens are processed following standard laboratory protocols and similar fixation times, we cannot exclude that minor variations could influence overall immunostaining intensity effecting labeling index measurements between cases.

In conclusion, we have shown that MAP2 is a useful adjunct to NeuN in the evaluation of neuronal preservation in CA4 in equivocal, indeterminate HS cases, suggesting that these cases more closely align with type 2 HS. Nevertheless, HS type in our series was not predictive of memory dysfunction or decline, which associated instead with multiple pathological factors, including neuronal and hippocampal pathway integrity, regenerative capacity and degenerative changes.

## Conflict of Interest

All authors have no conflict of interest to declare.

## Supporting information

Additional Supporting Information may be found in the online version of this article at the publisher's web‐site:

**Table S1.** Clinical data of temporal lobe epilepsy cases with no hippocampal sclerosis used for comparison for MAP2 and NeuN quantitative analysis.

**Table S2.** Antibodies and protocols for immunohistochemical studies.

**Table S3.** Results of statistical analysis between pathology measures and memory deficits.
