# Supplementary material for: Characterising subtypes of hippocampal sclerosis and reorganization: correlation with pre and postoperative memory deficit
Source: Brain Pathol. 2017 Apr 24;28(2):143–54. doi: 10.1111/bpa.12514 (PMC5893935; doi:10.1111/bpa.12514)
Supplement: Supplementary file 1 — Table S1. Clinical data of temporal lobe epilepsy cases with no hippocampal sclerosis used for comparison for MAP2 and NeuN quantitative analysis. [file BPA-28-143-s003.docx]

| **e/supplemental Table 1. Clinical data of temporal lobe epilepsy cases with no hippocampal sclerosis used for comparison for MAP2 and NeuN quantitative analysis.** | | | | | | | |
| --- | --- | --- | --- | --- | --- | --- | --- |
| CASE | GENDER | IPI | AGE ONSET EPILEPSY  (years) | Seizures type | AGE AT SURGERY  (years) | SIDE | FIRST YEAR OUTCOME |
| 1 | M | None | 15 | GS | 33 | R | Not seizure free |
| 2 | M | None | 14 | GS | 45 | R | Seizure free |
| 3 | M | Yes | 15 | SPS, CPS, SGS | 31 | L | Not seizure free |
| 4 | F | Yes | 9 | SPS, CPS, | 29 | R | Not seizure free |
| 5 | F | Yes; head injury | 23 | *PS | 44 | R | Not seizure free |
| 6 | M | Yes; head injury | 19 | SPS, CPS, SGS | 25 | R | Not seizure free |
| 7 | M | Yes | 22 | CPS | 24 | L | Seizure Free |
| GS = Generalised seizures, SGS = secondary generalised seizures, CPS = complex partial seizures, SPS = simple partial seizures (* Partial seizure type not further specified). | | | | | | | |
