# Supplementary material for: Characterising subtypes of hippocampal sclerosis and reorganization: correlation with pre and postoperative memory deficit
Source: Brain Pathol. 2017 Apr 24;28(2):143–54. doi: 10.1111/bpa.12514 (PMC5893935; doi:10.1111/bpa.12514)
Supplement: Supplementary file 2 — Table S2. Antibodies and protocols for immunohistochemical studies. [file BPA-28-143-s002.docx]

| **e/supplemental Table 2. Antibodies and protocols for immunohistochemical studies.** | | | | | | | |
| --- | --- | --- | --- | --- | --- | --- | --- |
| **Antibody** | **Description of target protein** | **Cell type / structures labelled** | **Type** | **Source** | **Antigen retrieval buffer** | **Time in buffer** | **Primary antibodies dilution and condition** |
| Anti-NeuN | Neuronal-specific nuclear protein | Neuronal nuclei | Monoclonal | Millipore, Watford, UK | Leica ER1 | 20 minutes | 1:2000; 15 minutes, RT |
| Anti-MAP2 | Microtubule associated protein 2 | Neuronal soma and dendrites | Monoclonal | Sigma Aldrich, Dorset, UK | H-3301 | 12 minutes | 1:1500; 1 hour, RT |
| Anti-Olig2 | Oligodendrocytes transcription factor 2 | Nuclei of oligodendrocytes and precursors | Polyclonal | Millipore, Watford, UK | Sodium Citrate pH 6.0 | 12 minutes | 1:250; overnight, 4ºC |
| Anti-Calbindin | Calcium binding protein | Soma and processes of granule cells and interneurons | Polyclonal | Swant, Marly, Switzerland | H-3300 | 12 minutes | 1:10,000; overnight, 4ºC |
| Anti-MCM2 | DNA replication licensing factor | Nuclei of proliferating cells and neural precursors | Monoclonal | BD Transduction Lab., Oxford, UK | H-3301 | 12 minutes | 1:900; overnight, 4ºC |
| Anti-Nestin | Intermediate filament protein | Soma and processes of immature neuro-glial cells | Monoclonal | Abcam, Cambridge, UK | H-3300 | 12 minutes | 1:1000; overnight, 4ºC |
| AT8 | Phosphorylated tau protein | Neurofibrillary tangles, neuropil threads | Monoclonal | Innogenetics, Gent, Belgium | Leica ER1 | 30 minutes | 1:1200; 20 minutes, RT |
| ZnT3 | Zinc transporter 3 | Mossy fibre axons | Polyclonal | Synaptic Systems, Germany | H-3300 | 12 minutes | 1:10,000; overnight, 4ºC |
| ER1, Bond citrate-based buffer (Leica, Milton Keynes, UK); H-3300, Vector’s citrate-based buffer pH 6.0 (Vector Lab, Peterborough, UK); H-3301, Vector’s Tris-based buffer pH 9.0 (Vector Lab, Peterborough, UK); MAP2, microtubule associated protein 2; MCM2, minichromosome maintenance marker 2; NeuN, neuronal nuclei; RT; room temperature; ZnT3, zinc transporter 3. | | | | | | | |
