# Supplementary material for: Characterising subtypes of hippocampal sclerosis and reorganization: correlation with pre and postoperative memory deficit
Source: Brain Pathol. 2017 Apr 24;28(2):143–54. doi: 10.1111/bpa.12514 (PMC5893935; doi:10.1111/bpa.12514)
Supplement: Supplementary file 3 — Table S3. Results of statistical analysis between pathology measures and memory deficits. [file BPA-28-143-s001.docx]

| **e/supplemental Table 3. Results of statistical analysis between pathology measures and memory deficits**. | | | | | |
| --- | --- | --- | --- | --- | --- |
| **Memory Domain** | **Region of interest** | **Immunomarker** | **Cases with deficit**  **Measurement (SD)**  **N= number of cases included in analysis** | **Cases without deficit**  **Measurement (SD)**  **N= number of cases included in analysis** | **Significance** |
| **Verbal memory deficit (moderate)** | DG | Olig2  MCM2  Calbindin* | 60/mm^2^ (27) N=22  8.1/mm^2^ (6.7) N=22  0.7 (0.87) N=22 | 71/mm^2^ (27) N=18  10.8/mm^2^ (17) N=18  1.2 (0.85) N=18 | p=0.7  p=0.35  p=0.18 |
| **Visual memory deficit (moderate)** | DG | Olig2  MCM2  Calbindin* | 65/mm^2^ (26) N=19  10/mm^2^ (18) N=19  0.89 (0.89) N=19 | 71/mm^2^ (29) N=21  9.2 / mm^2^ (7.2) N=21  1.09 (0.94) N=21 | p=0.68  p=0.18  p=0.5 |
| **Deficit in GNT (moderate)** | DG | Olig2  MCM2  Calbindin* | 73.9mm^2^ (27) N=13  10.7mm^2^ (8.6) N=13  0.84 (0.89) N=13 | 67 mm^2^ (23) N=21  9.5 mm^2^ (17) N=21  1.14 (0.8) N=21 | p=0.7  p=0.3  p=0.3 |
| **Verbal memory deficit (severe)** | DG | Olig2  MCM2  Calbindin* | 66/mm^2^ (9.3) N=6  12/mm^2^ (0.8) N=6  0.83 (0.98) N=6 | 70/mm^2^ (26) N=30  9.2/mm^2^ (15) N=30  1.1 (0.86) N=30 | p=0.8  p=0.1  p=0.6 |
| **Visual memory deficit (severe)** | DG | Olig2  MCM2  Calbindin* | 67/mm^2^ (19) N=3  6.6/mm^2^ (0.4) N=3  1 (1) N=3 | 70/mm^2^ (25) N=22  10/mm^2^ (17) N=22  1.1 (0.86) N=22 | p=0.9  p=0.7  p=0.9 |
| **Deficit in GNT (severe)** | DG | Olig2  MCM2  Calbindin* | 79/mm^2^ (31) N=9  9.2/mm^2^ (8) N=9  0.88 (0.92) N=9 | 65/mm^2^ (21) N=25  10/mm^2^ (16) N=25  1.08 (0.86) N=25 | p=0.2  p=0.78  p=0.6 |
| Results for Olig2, MCM2 and Calbindin quantitative analysis. The semi-quantitative score scales are detailed in supplementary methods and depicted in graphs (Fig. 2K,L). DG = dentate gyrus. | | | | | |
